# Combined IL-2 Immunocomplex and Anti-IL-5 mAb Treatment Expands Foxp3^+^ Treg Cells in the Absence of Eosinophilia and Ameliorates Experimental Colitis

**DOI:** 10.3389/fimmu.2019.00459

**Published:** 2019-03-14

**Authors:** Hirohito Abo, Kyle L. Flannigan, Duke Geem, Vu L. Ngo, Akihito Harusato, Timothy L. Denning

**Affiliations:** Center for Inflammation, Immunity & Infection, Institute for Biomedical Sciences, Georgia State University, Atlanta, Georgia

**Keywords:** IL-2, IL-5, Treg, eosinophil, ILC2, IBD

## Abstract

Interleukin (IL)-2 is expressed during T cell activation and induces the proliferation and differentiation of T cells. CD4^+^Foxp3^+^ regulatory T cells (Tregs) constitutively express the high affinity IL-2 receptor (CD25/IL-2Rα) and rapidly respond to IL-2 to elaborate numerous suppressive mechanisms that limit immune-mediated pathologies. Accumulating evidence supports the concept that an aberrant balance between Tregs and Teff contribute to the pathology of intestinal inflammation and that the IL-2/Treg axis is a potential pathway to exploit for the treatment of inflammatory bowel disease (IBD). Here, we show that treatment of mice with IL-2/IL-2 antibody (JES6-1) immunocomplex during DSS-induced colitis induced Foxp3^+^ Treg expansion, but also potently stimulated GATA3^+^ type 2 innate lymphoid cell (ILC2) proliferation and high-level expression of IL-5. Furthermore, IL-2/JES6-1 treatment resulted in massive eosinophil accumulation and activation in the inflamed colon, and afforded only modest protection from colitis. In light of these findings, we observed that combined IL-2/JES6-1 and anti-IL-5 mAb treatment was most effective at ameliorating DSS-induced colitis compared to either treatment alone and that this regimen allowed for Foxp3^+^ Treg expansion without concomitant eosinophilia. Collectively, our findings provide insight into how blockade of IL-5 may aid in optimizing IL-2 immunotherapy for the treatment of intestinal inflammation.

## Introduction

Interleukin (IL)-2 is a T cell growth factor that is essential for the proliferation and differentiation of T cells into effector and memory populations (1). These critical functions have led to the use of IL-2 in stimulating immune responses *in vivo*, particularly for anti-tumor immunotherapy and for boosting T cell numbers in AIDS patients (2, 3). However, the relatively high-doses of IL-2 required to induce beneficial responses *in vivo* are often accompanied by untoward side effects including vascular leak syndrome and hepatic and renal dysfunction, which have limited clinical use of high-dose IL-2 (4, 5). In addition to delivering IL-2 to augment immune activation, blockade of the high-affinity α chain of the IL-2 receptor (CD25) using the monoclonal antibody basilixumab, has also been employed to suppress organ transplantation rejection associated with IL-2 signaling (6). These clinical uses of IL-2 delivery or IL-2 receptor blockade to amplify or inhibit immune responses, respectively, fit with the well-defined immune stimulatory roles of IL-2.

Interestingly, IL-2 also plays a major role in the development, survival, expansion, and suppressive functions of a unique population of regulatory CD4^+^ T cells (Treg) that constitutively express high levels of CD25 and Foxp3 (7–18). Tregs play a vital role in negative regulation of immune-mediated inflammation in autoimmune and autoinflammatory disorders, cancer, infections, allergy, and intestinal inflammation. These cells also play key roles in suppressing metabolic inflammation and promoting tissue repair processes. Based on these suppressive functions the *in vivo* expansion of Foxp3^+^ Tregs has been explored as an avenue for treatment of inflammatory conditions. One method to expand Foxp3^+^ Tregs *in vivo* that has been pursued is the delivery of low-dose IL-2 either alone or complexed with antibodies. Low-dose IL-2 has been shown to preferentially expand Foxp3^+^ Tregs in part due to their constitutive CD25 expression as well as other cell-intrinsic factors (19). Treatment with low-dose IL-2 has shown promise in numerous inflammatory disorders including chronic graft vs.-host-disease (GVHD), allograft survival, systemic lupus erythematosus, and type I diabetes, among others (20, 21). Further, IL-2 can be complexed to antibodies that permit targeting to either Foxp3^+^ Tregs or effector T cells and some innate immune cell populations depending on the specific region of IL-2 the antibodies bind. For example, the anti-IL-2 antibody JES6-1 binding to IL-2 induces allosteric changes that permit preferential activation of CD25-expressing cells, such as Tregs, while a different anti-IL-2 antibody, S4B6, induces distinct conformational changes in IL-2 allowing for selective interaction with IL-2 receptor beta (IL-2Rβ/CD122) and expansion of CD8^+^ T cells and NK cells (22, 23). Consistent with the ability of IL-2/JES6-1 immunocomplexes to expand Foxp3^+^ Tregs *in vivo*, they have shown beneficial results in the treatment of several autoimmune and inflammatory diseases in mice (21). IL-2/JES6-1 immunocomplexes and low-dose IL-2 therapy are also stimulatory for group 2 innate lymphoid cells (ILC2s) that express CD25, which can contribute to IL-5 production and eosinophilia (24). Thus, the favorable effects of IL-2-mediated Foxp3^+^ Treg expansion and immune suppression may be tempered by simultaneous activation of immune stimulatory effector cells such as eosinophils.

Given the vast number of microbes in the intestine, tightly regulated immune responses are instrumental in the maintenance of gut homeostasis, and IL-2 and Foxp3^+^ Tregs play a vital function in this process (25–27). Upon bacterial colonization of the intestine in early life or upon colonization of germ-free mice with microbiota, IL-2 is rapidly induced and consequently drives the expansion of Foxp3^+^ Tregs that aids in establishing tolerance toward the microbiota (28, 29). The importance of IL-2 signaling and Foxp3^+^ Tregs in maintaining intestinal homeostasis is most evident in mice lacking IL-2 or Foxp3, which develop spontaneous intestinal inflammation (30, 31). Further, naive CD4^+^ T cells induce chronic colitis when transferred into immunodeficient mice in the absence of Foxp3+ Tregs (32). In this T cell transfer model, delivery of Foxp3^+^ Tregs can both prevent and treat disease (25). Based on these observations, *in vivo* expansion of Foxp3^+^ Tregs using IL-2 and other methods has been explored as a potential therapeutic for human inflammatory bowel disease (IBD) (33, 34).

In this report, we show that treatment of mice with IL-2/JES6-1 immunocomplex during DSS-induced colitis promoted Foxp3^+^ Treg expansion, but also potently stimulated GATA3^+^ group 2 innate lymphoid cell (ILC2) proliferation and high-level expression of IL-5 in the colon. Furthermore, IL-2/JES6-1 treatment resulted in massive eosinophil accumulation and activation in the inflamed colon, and afforded only modest protection from colitis. In light of these findings, we further demonstrated that combined IL-2/JES6-1 immunocomplex and anti-IL-5 mAb treatment was most effective at ameliorating DSS-induced colitis compared to either treatment alone and that this regimen allowed for Foxp3^+^ Treg expansion without concomitant eosinophilia. Collectively, our findings provide insight into how blockade of IL-5 may aid in optimizing IL-2 immunotherapy for the treatment of intestinal inflammation.

## Results

### IL-2/JES6-1 Immunocomplexes Induce Colonic Foxp3+ Treg Accumulation During DSS-Induced Intestinal Inflammation

**Figure 1:**
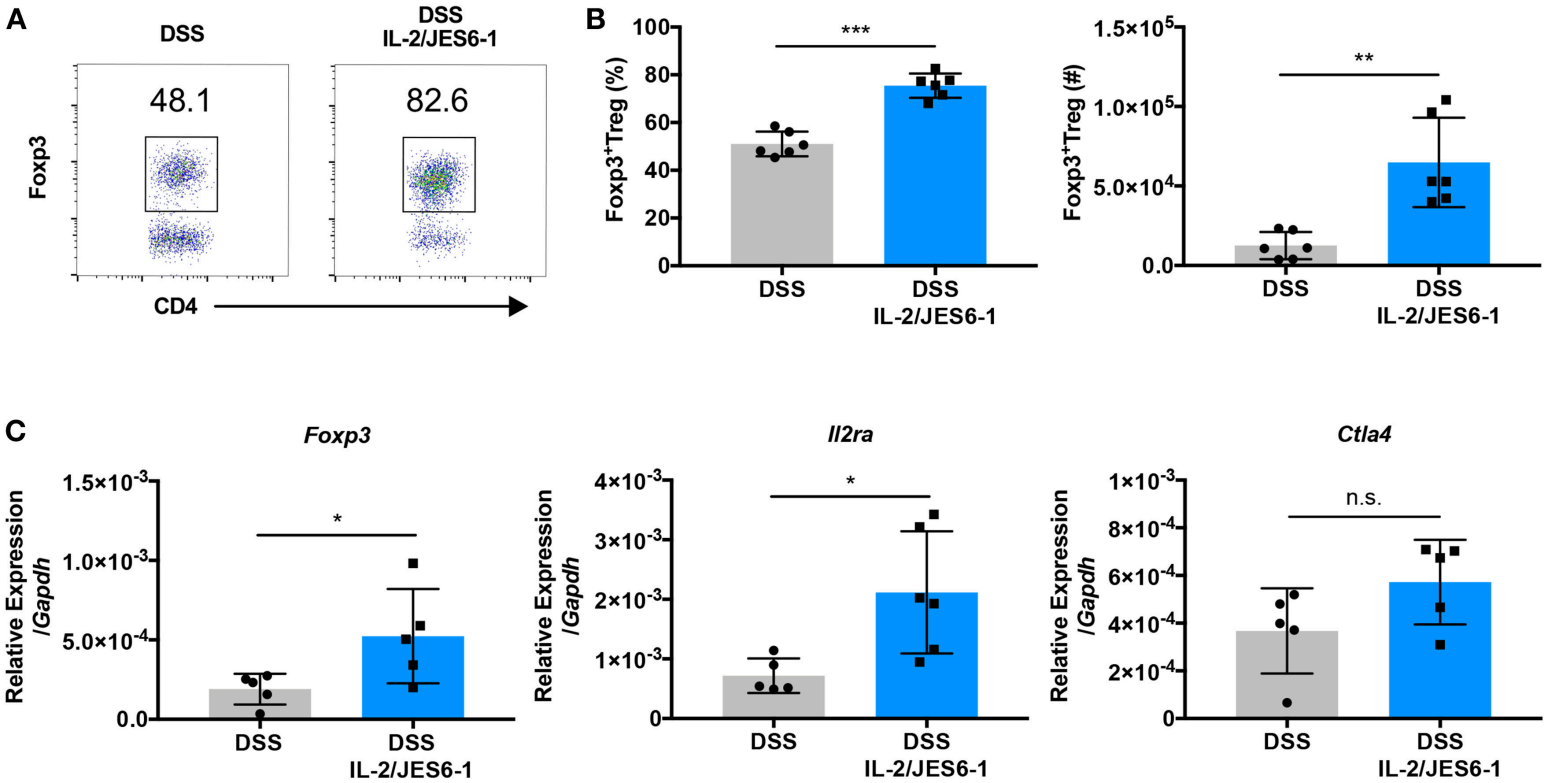
IL-2/JES6-1 immunocomplexes induce colonic Foxp3+ Treg accumulation during DSS-induced intestinal inflammation. WT mice were treated with 3% DSS in drinking water. At day 6, DSS water was replaced with normal water. IL-2/JES6-1 immunocomplexes were delivered every 2 days. CD4^+^Foxp3^+^Treg cells were analyzed by flow cytometry. Representative dot plots are shown in **(A)**. Frequency of CD4^+^Foxp3^+^ T cells are shown in **(A)** and total cell numbers of CD4+Foxp3+T cells are shown in **(B)**. **(C)** Expression of the Treg-related genes, *Foxp3, Cd25*, and *Ctla4* were analyzed by qPCR using total colonic tissue. Data are representative of two independent experiments with 4–6 mice/group. All data are presented as mean ± SEM; *P < 0.05, **P < 0.01, ***P < 0.001, one-way ANOVA with Tukey's multiple comparison test.

IL-2/JES6-1 immunocomplexes have been shown to induce rapid CD25^+^Foxp3^+^ Treg expansion in the spleen of mice and afford protection from dextran sodium sulfate (DSS)-induced colitis when delivered for one week prior to the initiation of DSS (22). In order to explore if IL-2 delivery can induce the accumulation of Foxp3^+^ Treg in the colons of mice during experimental colitis, we treated wild-type C57BL/6J mice with IL-2/JES6-1 immunocomplexes beginning at the time of DSS administration (day 0) and continuing every 2 days. At day 6, DSS was discontinued and colons were harvested at day 10. IL-2/JES6-1 immunocomplex treatment led to a significant increase in Foxp3^+^ Treg frequency and absolute cell number (Figures 1A,B). Additionally, the expanded Foxp3^+^ Treg population in IL-2/JES6-1 immunocomplex-treated mice coincided with increased colonic Foxp3, Il2ra, and Ctla4 mRNA expression (Figure 1C). Collectively, these data demonstrate that IL-2/JES6-1 immunocomplexes are capable to inducing robust Foxp3^+^ Treg accumulation in the colons of DSS-treated mice even when initiated at the same time as DSS dosing.

### Delivery of IL-2/JES6-1 Immunocomplexes to DSS-Treated Mice Drives Colonic Eosinophil Accumulation And Activation

**Figure 2:**
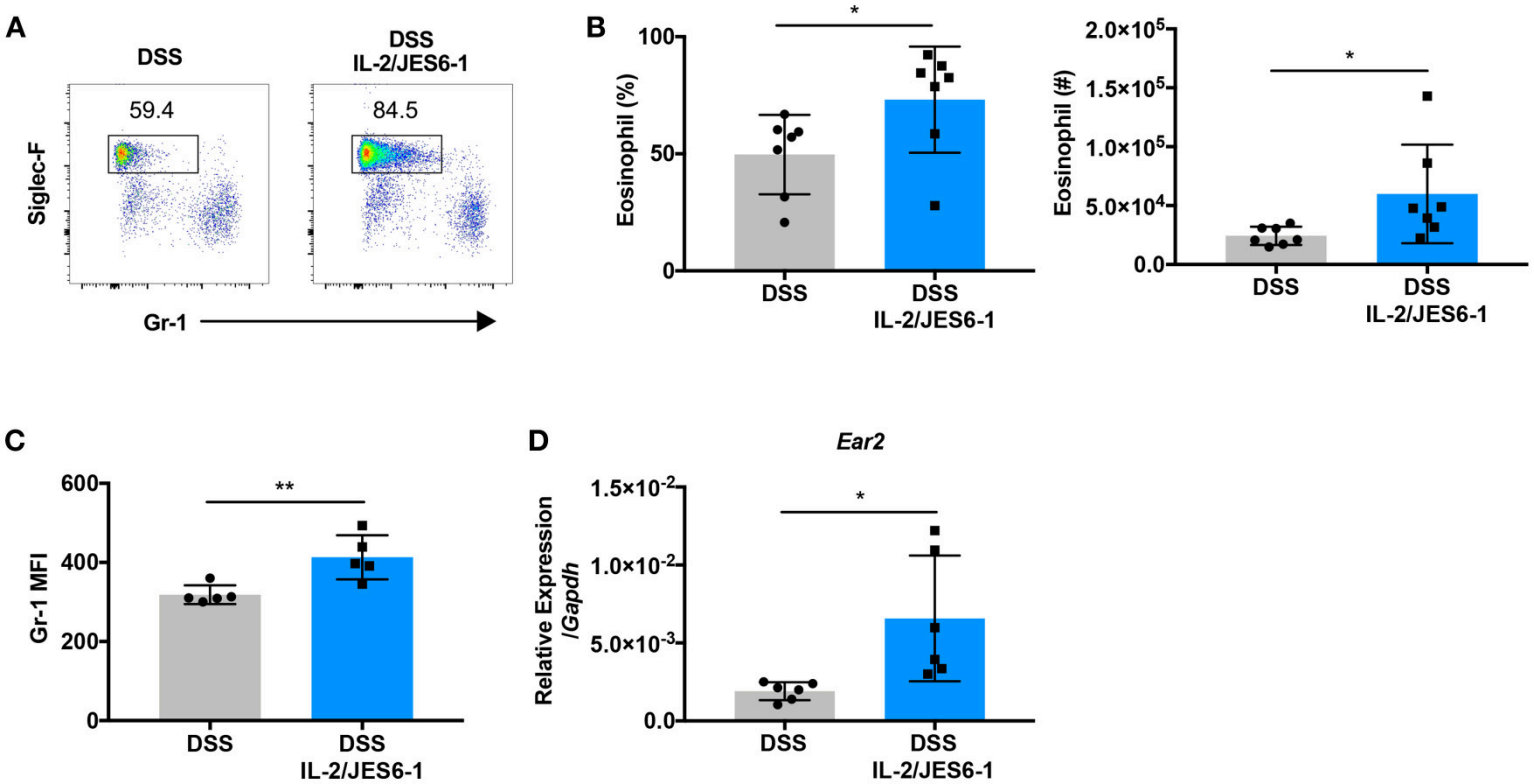
Delivery of IL-2/JES6-1 immunocomplexes to DSS-treated mice drives colonic eosinophil accumulation and activation. WT mice were treated with 3% DSS for 6 days and received normal water thereafter. IL-2/JES6-1 immunocomplexes were delivered every 2 days. On day 10, eosinophils were analyzed by flow cytometry by pre-gating on live, CD11b^+^ cells. Representative dot plots are shown in **(A)**, and the frequency and total cell numbers are shown in **(B)**. The mean fluorescence intensity (MFI) of Gr-1 staining in eosinophils is shown in **(C)** after pre-gating on live, CD11b^+^, Siglec-F^+^ cells. Expression of ECP (*Ear2*) was measured using qPCR **(D)**. Data are representative of two independent experiments with 5–7 mice/group. All data are presented as mean ± SEM; *P < 0.05, **P < 0.01, one-way ANOVA with Tukey's multiple comparison test.

Previous studies have reported IL-5-induced eosinophilia associated with IL-2/JES6-1 immunocomplex treatment in a murine model of dermatitis as well as in cancer patients that received high- or low-dose IL-2 immunotherapy (24). Therefore, we next explored whether IL-2/JES6-1 immunocomplexes affect colonic eosinophils during DSS-induced colitis. As shown in Figures 2A,B, IL-2/JES6-1 immunocomplex treatment led to a significant increase in Siglec-F^+^ eosinophil frequency and absolute cell number in the colons of DSS-treated mice. We next assessed whether IL-2/JES6-1 immunocomplex treatment led to eosinophil activation by analyzing Gr-1 expression, since previous studies have shown that eosinophils in the inflamed intestine show increased expression of intermediate, but not high, levels of Gr-1 (35). The Gr-1 antibody reacts with both Ly6C and Ly6G antigens that are also expressed by monocytes and neutrophils, respectively. Using both Gr-1 expression and side scatter (SSC) properties, these populations can be distinguished into Gr-1^lo−neg^SSC^hi^ eosinophils, Gr-1^hi^SSC^int^ neutrophils, and Gr-1^lo−int^SSC^lo^ monocytes (36). Upon activation, eosinophils can become Gr-1^int^SSC^hi^, and still be clearly distinguished from neutrophils and monocytes. Indeed, we observed that Siglec-F^+^ eosinophils in IL-2/JES6-1 immunocomplex-treated mice showed significantly higher mean fluorescence intensity (MFI) of Gr-1 when compared to non-treated controls (Figure 2C). We also investigated eosinophil cationic protein 2 (encoded by the *Ear2* gene) expression as another eosinophil activation marker (37). Consistent with enhanced Gr-1 expression, ear2 mRNA expression was increased by IL-2/JES6-1 immunocomplex administration to DSS-treated mice (Figure 2D). These data indicate that IL-2/JES6-1 immunocomplexes can potently induce eosinophil accumulation and activation in the inflamed intestine during experimental colitis, which may be an unwanted side effect of IL-2-based immunotherapy forintestinal inflammation.

### IL-2/JES6-1 Immunocomplex Administration Promotes Intestinal ILC2 Expansion and IL-5 Expression

**Figure 3:**
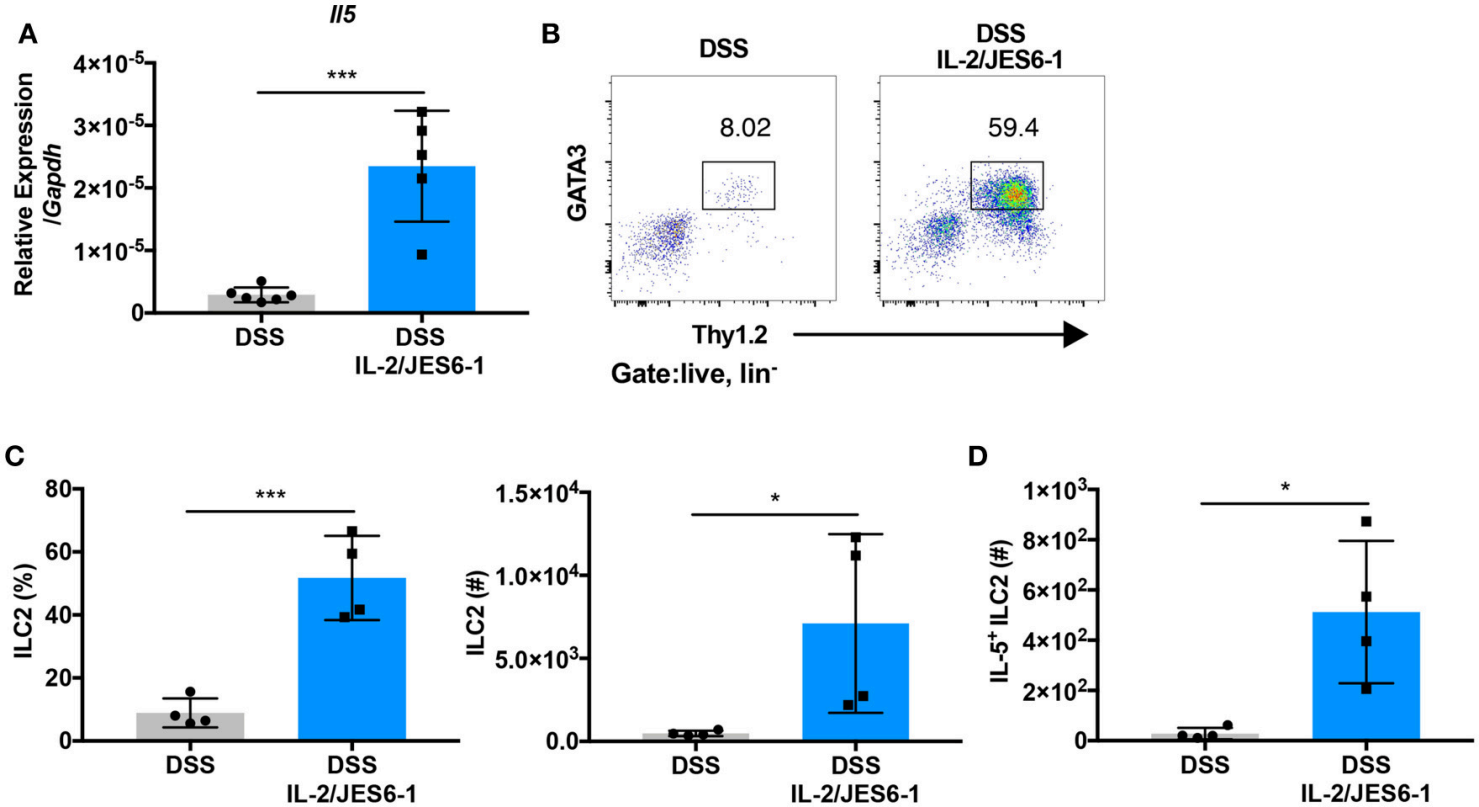
IL-2/JES6-1 immunocomplex administration promotes intestinal ILC2 expansion and IL-5 expression. WT mice were treated with 3% DSS for 6 days and received normal water thereafter. IL-2/JES6-1 immunocomplexes were delivered every 2 days. IL-5 mRNA expression in total colon tissue was analyzed by quantitative real-time PCR **(A)**. LPL from each mouse were restimulated by PMA/ionomycin and ILC2 cells were analyzed by flow cytometry. Represent dot plots are shown in **(B)** and the frequency and total cell numbers are shown in **(C)** after pre-gating on live, lin- cells. IL-5+ILC2 were analyzed by flow cytometry **(D)**. Data are representative of two independent experiments with 4–6 mice/group. All data are presented as mean ± SEM; *P < 0.05, ***P < 0.001, one-way ANOVA with Tukey's multiple comparison test.

To determine the mechanism of colonic eosinophil accumulation induced by IL-2/JES6-1 immunocomplex treatment in DSS-treated mice, we next investigated group 2 innate lymphoid cells (ILC2s). ILC2s are defined as lin^−^CD127^+^CD90^+^ and express the transcription factor GATA3 (38). Several studies have reported that ILC2s express CD25 and respond to IL-2 stimulation by proliferating and elaborating type 2 cytokine expression (39, 40). Additionally, a recent report showed that IL-5-producing ILC2s control eosinophilia induced by IL-2 therapy in humans and mice (24). Upon analysis of DSS-treated mice, we observed that administration of IL-2/JES6-1 immunocomplexes induced a significant increase in IL-5 mRNA expression in total colonic tissue (Figure 3A). Furthermore, IL-2/JES6-1 immunocomplexes significantly increased lin^−^CD90.2^+^GATA3^+^ ILC2 frequency and absolute cell number (Figures 3B,C). IL-5 expressing ILC2s were also significantly increased in IL-2/JES6-1 immunocomplex-treated mice (Figure 3D), as was IL-13 mRNA expression (Supplementary Figure 1A) and IL-13 expressing ILC2s (Supplementary Figure 1B). While Ccl11 mRNA expression was significantly induced following DSS, IL-2/JES6-1 immunocomplex treatment did not significantly augment expression levels (Supplementary Figure 1C). Together, these observations suggest that eosinophil accumulation and activation induced by IL-2/JES6-1 immunocomplex treatment was strongly associated with IL-5- and IL-13-producing ILC2 induction.

### Combined Delivery of IL-2/JES6-1 Immunocomplexes and Anti-IL-5 Mab Ameliorates DSS-Induced Colitis

**Figure 4:**
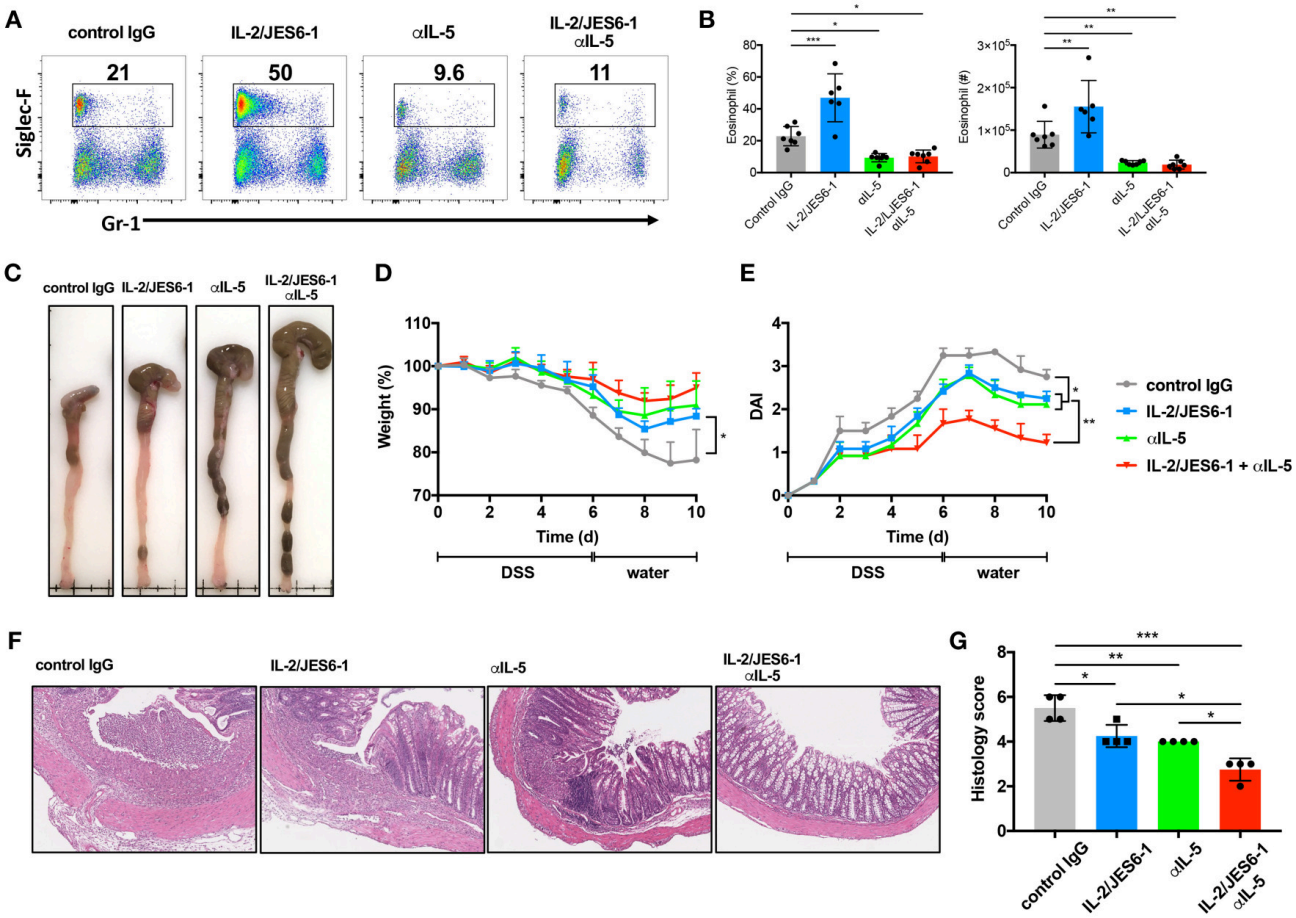
Combination of IL-2/JES6-1 immunocomplexes and anti-IL-5 mAb effectively ameliorate DSS-induced colitis. WT mice were treated with 3% DSS for 6 days and received normal water thereafter. IL-2/JES6-1 immunocomplex were delivered every 2 days and anti-IL-5 mAb was delivered every day. Eosinophils (Siglec-F^+^) were analyzed by flow cytometry **(A)** and the frequency and total cell numbers are shown in **(B)**. Representative images of colons from indicated conditions are shown in **(C)**. Weight loss **(D)** and disease activity index (DAI) **(E)** were monitored every day. Representative hematoxylin/eosin stained colon sections are shown in **(F)** and histology scoring of individual colon sections are shown in **(G)**. Data are representative of two independent experiments with 4 mice/group. All data are presented as mean ± SEM; *P < 0.05, **P < 0.01, and ***P < 0.001, one-way ANOVA with Tukey's multiple comparison test.

In light of our observations that IL-2/JES6-1 immunocomplexes induce Foxp3^+^ Tregs, but also IL-5-producing ILC2s and activated eosinophils during DSS-induced colitis, we next attempted a combined approach to optimally ameliorate colitis by expanding colonic Foxp3+ Tregs via delivery of IL-2/JES6-1 immunocomplexes while simultaneously blocking eosinophil accumulation and activation by using anti-IL-5 monoclonal antibodies (mAb). We first assessed whether anti-IL-5 mAb treatment was able to prevent colonic eosinophilia in our experimental model. Indeed, while IL-2/JES6-1 immunocomplexes increased colonic eosinophil frequency and absolute cell number in DSS-treated mice, this effect could be prevented by co-administration of anti-IL-5 mAb (Figures 4A,B). Of note, administration of anti-IL-5 mAb alone was also able to significantly reduce colonic eosinophils when compared to control IgG administration to DSS-treated mice. Similarly, IL-2/JES6-1 immunocomplexes increased colonic eosinophil activation as evidenced by increased frequencies and absolute cell numbers of cells expressing intermediate, but not high, levels of Gr-1, and this effect could be prevented by co-administration of anti-IL-5 mAb (Supplementary Figures 2A,B). We next examined the effects of these 4 different treatments (control IgG, IL-2/JES6-1 immunocomplexes alone, anti-IL-5 mAb alone, and IL-2/JES6-1 immunocomplexes + anti-IL-5 mAb) on DSS-induced colitis and recovery. While the IL-2/JES6-1 immunocomplex group and the anti-IL-5 mAb group each displayed improvement in disease outcome compared the control IgG treated group, combined delivery of IL-2/JES6-1 immunocomplexes + anti-IL-5 mAb afforded the most significant protection as measured by reduced colonic shortening (Figure 4C), weight loss (Figure 4D), and disease activity index (DAI; Figure 4E). Consistent with these observations, combined delivery of IL-2/JES6-1 immunocomplexes + anti-IL-5 mAb also provided optimal reduction in pathological tissue inflammation (Figure 4F) and histology score (Figure 4G). As summarized in Supplementary Figure 3, these data collectively demonstrate that combined administration of IL-2/JES6-1 immunocomplexes and anti-IL-5 mAb, which allows for Foxp3^+^ Treg expansion in the absence of eosinophilia, is highly effective at ameliorating DSS-induced colitis compared to the use of IL-2/JES6-1 immunocomplexes or anti-IL-5 mAb alone.

## Discussion

In this study, we provide evidence demonstrating that combined delivery of IL-2 immunocomplexes and anti-IL-5 mAb is highly effective at expanding Foxp3^+^ Treg cells in the absence of eosinophilia and ameliorating DSS-induced colitis in mice. While evidence strongly suggests that Tregs are instrumental in establishing and maintaining gut homeostasis, an effective approach to expand these cells *in vivo* for the treatment of intestinal inflammation, without undesired side effects, has proven challenging (21, 33, 34). A previous report elegantly demonstrated that IL-2/JES6-1 immunocomplexes are effective at expanding Foxp3+ T cells in the spleens of mice and when delivered for 1 week prior to the initiation of DSS were able to lessen colitis (22). Our data confirms and extends these findings by showing that administration of IL-2/JES6-1 immunocomplexes at the time of initiating DSS treatment is sufficient to expand colonic Foxp3^+^ Tregs and ameliorate colitis.

Interestingly, we noted that IL-2/JES6-1 immunocomplex delivery to DSS-treated mice also potently expanded IL-5 producing ILC2s and eosinophils in the inflamed colon, which is consistent with observations in other tissues (24, 40). These data suggested that accumulation and activation of eosinophils may have been impairing the beneficial effects of Foxp3^+^ Treg expansion induced by IL-2/JES6-1 immunocomplexes. Consistent with this concept, activated eosinophils have been associated with human IBD (41–44) and DSS-induced histopathology is attenuated in mice deficient in eosinophils (45–47), although eosinophils appear to play a dual role in the intestine and can provide beneficial effects (48, 49). Given the potent role for IL-5 in driving eosinophil accumulation, blockade of IL-5 has also been shown to reduce intestinal eosinophilia and modestly ameliorate experimental colitis in some models (35), yet not in others (50, 51). Indeed, two previous studies analyzing DSS-induced colitis using IL-5-deficient mice (50, 51) concluded that IL-5 alone plays a minor role in the DSS model, whereas we observed a modestly more robust effect in our study. These modest effects of IL-5 deficiency on DSS colitis may be due to eosinophils providing both pro- and anti-inflammatory functions during intestinal inflammation (52), as well as the use of IL-5-deficient mice in these studies and antibody-mediated IL-5 neutralization in our study. In response to IL-2/JES6-1 immunocomplex administration and increased levels of IL-5, the augmented number and activation status of eosinophils may tip the balance in the pro-inflammatory direction, which may explain our observed beneficial effect of combined IL-2/JES6-1 immunocomplex and IL-5 blockade in the DSS model of colitis. It is noteworthy that the acute DSS model of colonic damage and repair predominantly involves innate immune activation and whether this combined treatment strategy will also be effective in chronic, T-cell dependent models of intestinal inflammation remains to be investigated.

The expansion of ILC2s by IL-2 immunocomplexes has been well-documented and consistent with the high expression of CD25 on ILC2s (21, 24, 40). However, whether ILC2 activation is beneficial or deleterious during intestinal inflammation is still being actively unraveled. Recent reports have shown that these cells are involved in mounting type 2 immune responses to helminthic infections of the gastrointestinal tract and coordinate with tuft cells to elaborate an IL-25-ILC2-IL-13 immune circuit that promotes intestinal defense and remodeling (53–56). During DSS-induced colitis however, the function of ILC2s is less clear. Gut-associated ILC2s have been reported to secrete the growth factor amphiregulin which can limit intestinal inflammation and promote tissue repair processes (57). As with eosinophils, ILC2 expansion by IL-2 immunocomplexes may alter the normal function of these cells. Clearly, additional studies are warranted to fully understand how IL-2 delivery regulates ILC2s as well as eosinophils.

Overall, further understanding of how to exploit IL-2 signaling to enhance the development, function, and antigen specificity of Tregs, without unwelcome side effects, is an active area of investigation with strong translational potential for many inflammatory diseases (34). IL-2/JES6-1 immunocomplexes have been successfully used to expand Foxp3^+^ Treg cells in mice and these findings have led to the development of an anti-human IL-2 mAbs that selectively expands Tregs in humans (21, 34, 58). Recently, a novel anti-IL-2 antibody, F5111.2, was developed that stabilizes IL-2 in a conformation that leads to the selective expansion of Tregs. Complexing of F5111.2 with human IL-2 led to the remission of type 1 diabetes and reduced disease severity in mouse models of graft-vs.-host disease and experimental autoimmune encephalomyelitis (59). Another clinically attractive approach for preferentially expanding Tregs *in vivo* is to directly modulate IL-2 itself to allow for optimal conformation for binding to CD25 (34). These so-called IL-2 “muteins,” like IL-2 immunocomplexes, may also lead to ILC2 expansion, IL-5 production, and eosinophilia. The data presented here suggests that these approaches for IL-2 driven Treg expansion *in vivo* may be further optimized by blocking IL-5 and limiting eosinophil activation.

In the present study we assessed IL-5 induction as one unwarranted side-effect of IL-2 immunocomplex treatment, however, it is important to note that stimulation of ILC2s with IL-2 or IL-2 immunocomplexes also induces other type 2 cytokines that may contribute to intestinal inflammation, including IL-9 (60) and IL-13 (40). Interestingly, both IL-9 and IL-13 can contribute to the pathogenesis of experimental models of colitis and human inflammatory bowel disease (IBD) (61, 62) and are known inducers of the eosinophil chemokine Ccl11 (63, 64). These findings may thus provide a framework for future investigations into whether blockade of IL-5, IL-9, IL-13, and/or Ccl11 may enhance the effectiveness of IL-2 immunocomplex treatment during intestinal inflammation as well as other inflammatory disease settings.

## Materials and Methods

### Mice

C57BL/6J mice were obtained from the Jackson Laboratory and maintained in specific pathogen-free conditions. In all experiments, sex-matched mice were used at 7–8 weeks of age. Animal procedures were approved by the Institutional Animal Care and Use Committee of Georgia State University.

### DSS Model of Colitis

Mice were treated with 3% (wt/vol) DSS (MP Biomedicals; molecular weight: 36,000–50,000) in their drinking water for 6 days and then DSS was replaced with normal water. Mice receiving DSS were monitored daily for weight change and disease index activity (DAI).

### Flow Cytometry

Fluorescent dye-labeled antibodies specific for CD4, CD25, CD11b, Gr-1 (clone RB6-8C5), Foxp3, GATA3, B220, NK1.1, CD19, CD3, IL-5, and IL-13 were purchased from eBioscience and Biolegend. Fc block (2.4G2) was purchased from BD. Dead cells were stained by fixable aqua dead cell staining kit. Intracellular staining for Foxp3 and GATA3 was performed by Intracellular Fixation and Permeabilization Buffer Set (eBioscience). Intracellular staining of IL-5 was performed after restimulation with PMA, ionomycin and brefeldin A for 5 h. Restimulated cells were treated with Intracellular Fixation and Permeabilization Buffer Set (eBioscience) and stained with IL-5 antibodies. Flow cytometric analysis was performed on a Beckman Coulter Cytoflex flow cytometer and analyzed by Flowjo software (Tree Star, Ashland, OR).

### Isolation of Colonic Lamina Propria Cells From Large Intestine

Colon tissues were cut into 0.5 cm pieces and transferred into 50 mL conical tubes. Then tubes were shaken at 250 rpm for 20 min at 37°C in Hanks' balanced salt solution supplemented with 5% FBS with 5 mM EDTA. This process was repeated twice. Cell suspensions were passed through a cell strainer and remaining colon tissues were washed and minced, transferred to 50 mL conical tubes and shaken for 10 min at 37°C in Hanks' balanced-salt solution supplemented with 5% FBS and type VIII collagenase (1 mg/mL). Cell suspensions were passed through a cell strainer and pelleted by centrifugation at 300 g.

### *In vivo* Administration of IL-2/JES6-1 Immunocomplexes and Anti-IL-5 mAb

IL-2/JES6-1 immunocomplexes were prepared by pre-incubating recombinant IL-2 (PeproTech) with anti-IL-2 mAb (JES6-1; Bioxcell) at a 2:1 cytokine:antibody molar ratio for 30 min at room temperature (23, 65). Mice were injected with anti-IL-5 antibodies (200 μg) and/or IL-2/JES6-1 immunocomplexes (1 mg every 2 days) by i.p injection.

### RNA Isolation and Real-Time PCR

Total RNA was isolated from colon tissue using the Qiagen RNeasy Mini Kit, according to the manufacturer's protocols with on-column DNase digestion using the RNase Free DNase set. cDNA was generated using Hi-capacity cDNA Reverse Transcription Kit (Applied Biosystems) according to manufacturer's protocols. qPCR was performed with SYBR Green Master Mix (BioRad) on Step One Plus real time PCR system (Applied Biosystems), and gene specific primers.

*Il5* F: 5′-CGCTCACCGAGCTCTGTTG-3′

*Il5* R: 5′-CCAATGCATAGCTGGTGATTTTT-3′

*Foxp3* F: 5′-CACCCAGGAAAGACAGCAACC-3′

*Foxp3* R: 5′-GCAAGAGCTCTTGTCCATTGA-3′

*Gapdh* F: 5′-TGGCAAAGTGGAGATTGTTGCC-3′

*Gapdh* R: 5′-AAGATGGTGATGGGCTTCCCG-3′

*Ctla4* F: 5′-TGTTGACACGGGACTGTACCT-3′

*Ctla4* R: 5′-CGGGCATGGTTCTGGATCA-3′

*Il2ra* F: 5′-CCACCACAGACTTCCCACAA-3′

*Il2ra* R: 5′-CCATCTGTGTTGCCAGGTGA-3′

*Ear2* F: 5′-ACCAGTCGGAGGAGAACACC-3′

*Ear2* R: 5′-CAAAGGTGCAAAGTGCTGGC-3′

*Ccl11* F: 5′-AGAGCTCCACAGCGCTTCTATT-3′

*Ccl11* R: 5′-GGTGCATCTGTTGTTGGTGATT-3′

### Histology

Colon tissues were fixed in 10% neutral buffer formalin and embedded in paraffin. Paraffin embedded tissue sections were stained using hematoxylin/eosin. The score of inflammation and epithelial damage was graded in a blinded manner using a scale from 0 to 3 for each parameter.

### Statistics

All statistical analyses were performed with GraphPad Prism software, version 7 (GraphPad Software). One-way ANOVA and Tukey's Multiple Comparison Test or Student's *t*-test were used to determine significance. *P* < 0.05 were considered significant. ^*^P < 0.05, ^**^P < 0.01, ^***^P < 0.001.

## Data Availability

All datasets generated for this study are included in the manuscript and/or the supplementary files.

## Author Contributions

HA and TD conceived the idea for this project and designed the experiments. HA performed all of the experiments and analyzed the data. KF and DG performed critical preliminary experiments. VN and AH provided technical assistance. HA and TD wrote the manuscript.

### Conflict of Interest Statement

The authors declare that the research was conducted in the absence of any commercial or financial relationships that could be construed as a potential conflict of interest.

## Supplementary Material

Supplementary Figure 1 IL-2/JES6-1 immunocomplex administration induces expansion of IL-13-producing intestinal ILC2s. WT mice were treated with 3% DSS for 6 days and received normal water thereafter. IL-2/JES6-1 immunocomplexes were delivered every 2 days. IL-13 mRNA expression in total colon tissue was analyzed by quantitative real-time PCR **(A)**. LPL from each mouse were restimulated by PMA/ionomycin and IL-13+ ILC2s total cell numbers were analyzed by flow cytometry after pre-gating on live, lin- cells **(B)**. Ccl11 mRNA expression in total colon tissue was analyzed by quantitative real-time PCR **(C)**. All data are presented as mean ± SEM; ^*^*P* < 0.05, ^**^*P* < 0.01.

Supplementary Figure 2 Anti-IL-5 mAb treatment efficiently prevents IL-2/JES6-1 immunocomplex-induced eosinophil activation during DSS-induced colitis. WT mice were treated with 3% DSS for 6 days and received normal water thereafter. IL-2/JES6-1 immunocomplexes were delivered every 2 days and anti-IL-5 mAb was delivered every day. Siglec-F^+^ populations were gated as Gr-1 low, int, and hi. Representative dot plots are shown in **(A)**. Frequency and cell numbers of each population are shown in **(B)**. All data are presented as mean ± SEM; ^*^*P* < 0.05, ^**^*P* < 0.01, ^***^*P* < 0.001.

Supplementary Figure 3 Model for combined effects of IL-2/JES6-1 immunocomplex and anti-IL-5 treatment during DSS colitis. IL-2/JES6-1 immunocomplexes during DSS-induced colitis induce Foxp3^+^ Treg expansion, but also potently stimulates GATA3^+^ILC2 proliferation and expression of IL-5, leading to eosinophil accumulation and activation in the inflamed colon. Combined IL-2/JES6-1 and anti-IL-5 mAb treatment permits Foxp3+ Treg expansion in the absence of associated eosinophilia and is effective at ameliorating DSS-induced colitis.
