# Supplementary figures and images for: Combined IL-2 Immunocomplex and Anti-IL-5 mAb Treatment Expands Foxp3+ Treg Cells in the Absence of Eosinophilia and Ameliorates Experimental Colitis
Source: Front Immunol. 2019 Mar 14;10:459. doi: 10.3389/fimmu.2019.00459 (PMC6428029; doi:10.3389/fimmu.2019.00459)

# Supplementary Figure 1

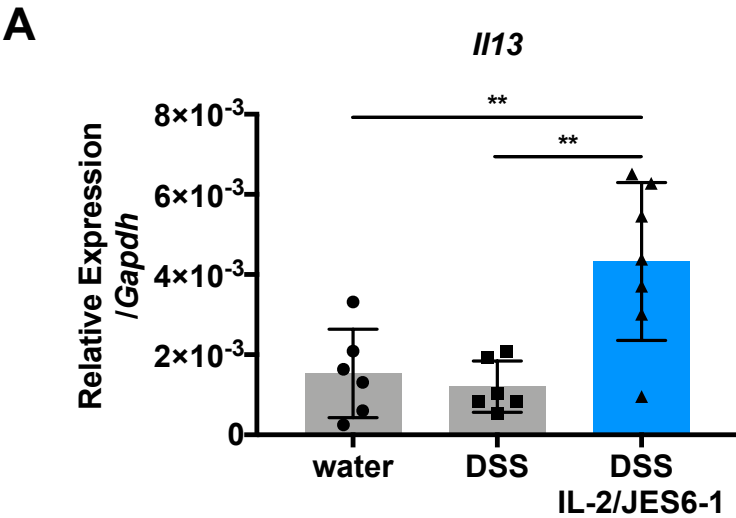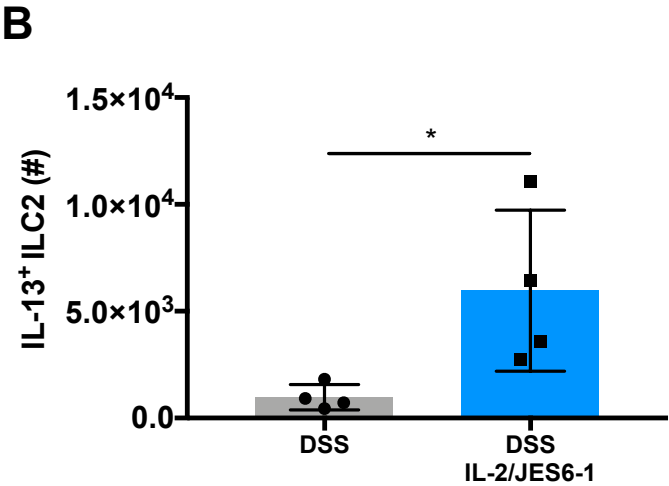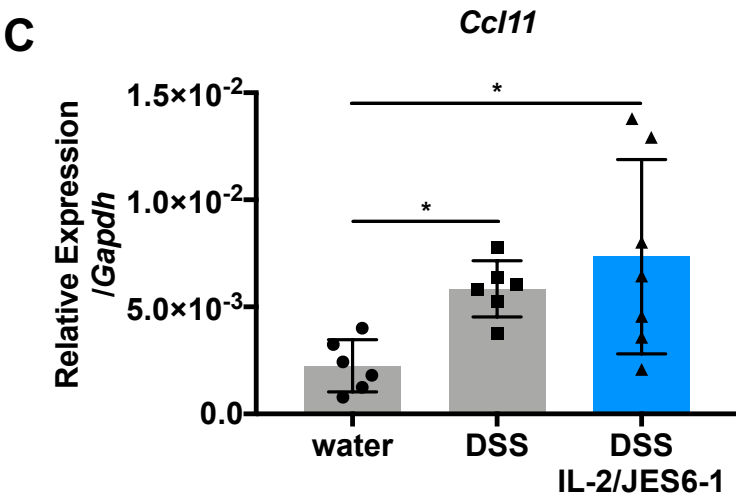

## Supplementary Figure 2

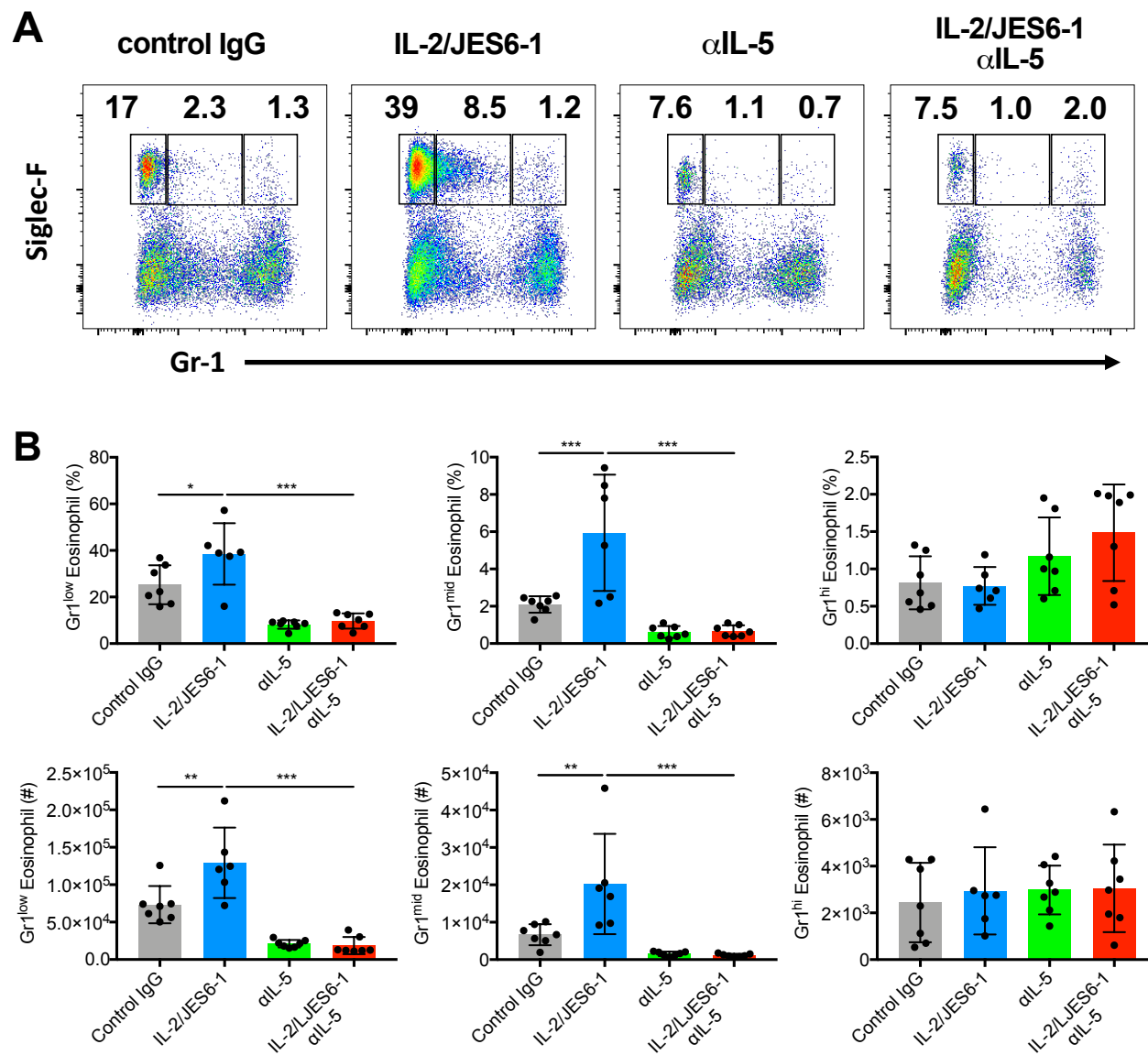

Supplementary Figure 3

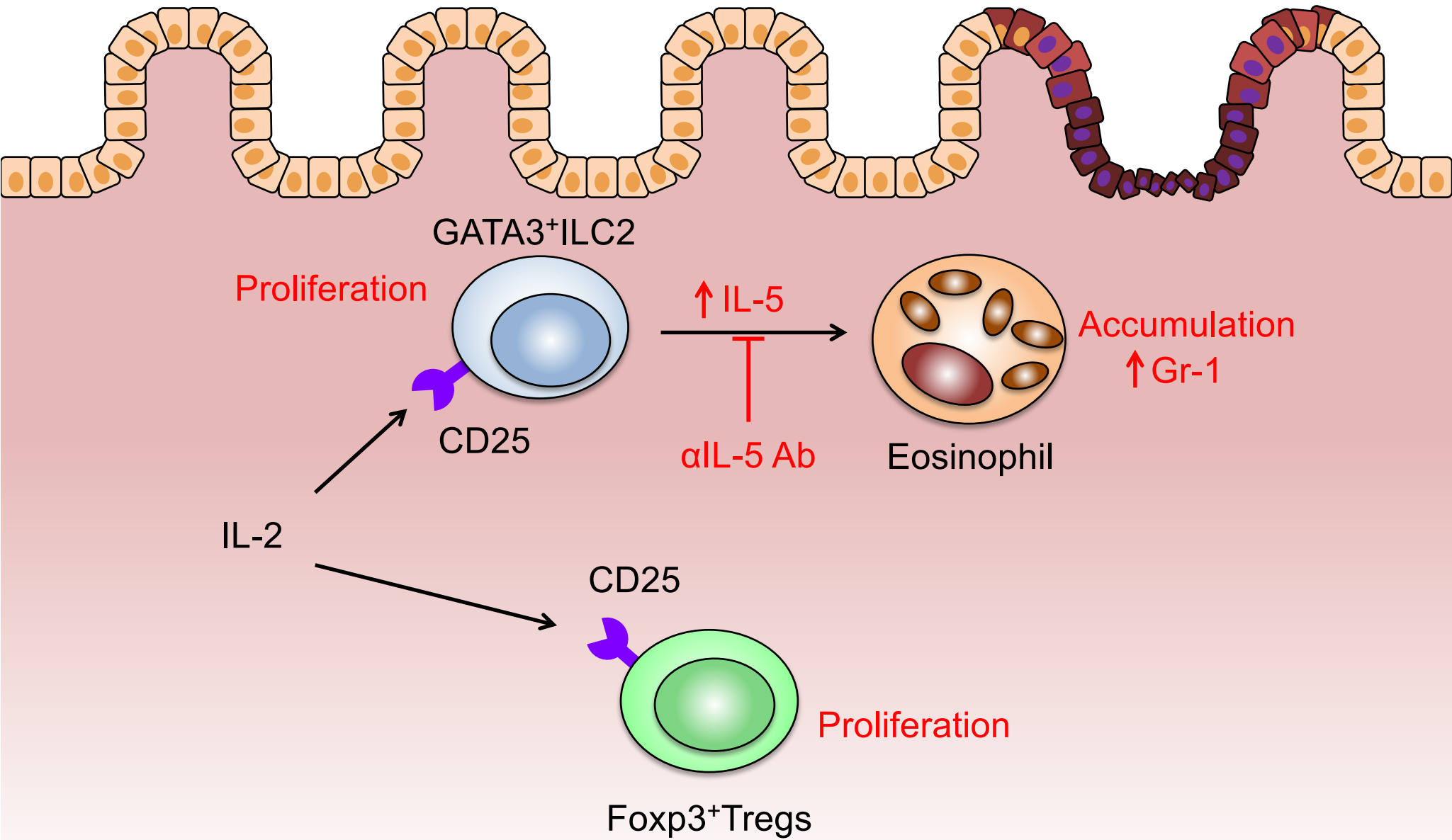

Supplement: Supplementary Figure 1 — IL-2/JES6-1 immunocomplex administration induces expansion of IL-13-producing intestinal ILC2s. WT mice were treated with 3% DSS for 6 days and received normal water thereafter. IL-2/JES6-1 immunocomplexes were delivered every 2 days. IL-13 mRNA expression in total colon tissue was analyzed by quantitative real-time PCR (A). LPL from each mouse were restimulated by PMA/ionomycin and IL-13+ ILC2s total cell numbers were analyzed by flow cytometry after pre-gating on live, lin- cells (B). Ccl11 mRNA expression in total colon tissue was analyzed by quantitative real-time PCR (C). All data are presented as mean ± SEM; *P < 0.05, **P < 0.01. [file Data_Sheet_1.PDF]
